# The impact of post-traumatic stress on quality of life and fatigue in women with Gulf War Illness

**DOI:** 10.1186/s40359-022-00752-5

**Published:** 2022-02-25

**Authors:** Nandan Shastry, Esha Sultana, Mary Jeffrey, Fanny Collado, Jeffrey Kibler, Christian DeLucia, Mary Ann Fletcher, Nancy Klimas, Travis J. A. Craddock

**Affiliations:** 1 Institute for Neuro-Immune Medicine, Nova Southeastern University, Fort Lauderdale, FL USA; 2 Department of Psychology and Neuroscience, Nova Southeastern University, Fort Lauderdale, FL USA; 3 Geriatric Research, Education, and Clinical Center, Miami Veterans Affairs Medical Center, Miami, USA; 4 Department of Clinical and School Psychology, Nova Southeastern University, Fort Lauderdale, FL USA; 5 Department of Clinical Immunology, Nova Southeastern University, Fort Lauderdale, FL USA; 6 Department of Computer Science, Nova Southeastern University, Fort Lauderdale, FL USA

**Keywords:** Gulf War Illness, Post-traumatic stress disorder, Co-morbid conditions, Hierarchical regression, Sub-typing

## Abstract

**Background:**

Gulf War Illness (GWI) is a chronic, multi-symptomatic disorder characterized by fatigue, muscle pain, cognitive problems, insomnia, rashes, and gastrointestinal issues affecting an estimated 30% of the ~ 750,000 returning military Veterans of the 1990–1991 Persian Gulf War. Female Veterans deployed to combat in this war report medical symptoms, like cognition and respiratory troubles, at twice the rate compared to non-deployed female Veterans of the same era. The heterogeneity of GWI symptom presentation complicates diagnosis as well as the identification of effective treatments. This is exacerbated by the presence of co-morbidities. Defining subgroups of the illness may help alleviate these complications. One clear grouping is along the lines of gender. Our aim is to determine if women with GWI can be further subdivided into distinct subgroups based on post-traumatic stress disorder (PTSD) symptom presentation.

**Methods:**

Veterans diagnosed with GWI (*n* = 35) and healthy sedentary controls (*n* = 35) were recruited through the Miami Veterans Affairs Medical Health Center. Symptoms were assessed via the RAND short form health survey, the multidimensional fatigue inventory, and the Davidson trauma scale. Hierarchal regression modeling was performed on measures of health and fatigue with PTSD symptoms as a covariate. This was followed by univariate analyses conducted with two separate GWI groups based on a cut-point of 70 for their total Davidson trauma scale value and performing heteroscedastic t-tests across all measures.

**Results:**

Based on the distinct differences found in PTSD symptomology regarding all health and trauma symptoms, two subgroups were derived within female GWI Veterans. Hierarchical regression models displayed the comorbid effects of GWI and PTSD, as both conditions had measurable impacts on quality of life and fatigue (*ΔR*^2^ = 0.08–0.672), with notable differences in mental and emotional measures. Overall, a cut point analysis indicated poorer quality of life and greater fatigue within all measures for women with GWI and PTSD symptoms in comparison to those women with GWI without PTSD symptoms and healthy controls.

**Conclusions:**

Our current findings support the understanding that comorbid symptoms of GWI and PTSD subsequently result in poorer quality of life and fatigue, along with establishing the possibility of varying clinical presentations.

## Background

Gulf War Illness (GWI) continues to negatively impact at least 25 percent of the 700,000 deployed US military personnel to the 1990–1991 Gulf War [1]. Of those 700,000 deployed nearly 50,000 were women [2]. GWI is a chronic condition expressed as a combination of fatigue, pain, headache, difficulty concentrating, memory loss, sleep disturbance, respiratory issues, gastrointestinal problems, and skin rash [3]. Identifying the pathology of GWI is complicated by the disease’s interaction with multiple systems of the body including the central nervous system, autonomic nervous system, immune system, and endocrine system (i.e., hypothalamic–pituitary–adrenal [HPA] axis, hypothalamic–pituitary–adrenal [HPG] axis [4]; Research Advisory Committee on Gulf War Veterans’ Illnesses [1]. The etiology of GWI is further complicated when considering the role of gender or how the pathobiological expression of GWI may differ between male and female Veterans [1].

The Gulf War had the largest proportion of women serving in a military zone, comprising of 7 percent of those deployed to the Gulf War. Furthermore, military roles for women expanded which increased their exposure to combat and neurotoxicants [5]. However, research pertaining to woman health issues is relatively sparse for female Veterans of the Gulf War [6] and predominately focused on reproductive health (i.e., stillbirths, pregnancies, birth defects).

Early research investigating female and male Veterans of the Gulf War era and Gulf War in specific provided initial support for a differential expression by gender regarding health outcomes. Like the general population, incidence rates of multiple sclerosis were three times higher in female Veterans in comparison to male Veterans who served in the Gulf War era from 1990 to 2007 [7]. Similarly, female Gulf War era Veterans were more likely to report osteoporosis, bipolar disorder, depression, irritable bowel syndrome, migraines, asthma, and thyroid issues in comparison to their male counterparts [8]. Female Veterans of the Gulf War era also report poorer health and medical diagnoses (outside of diabetes and cardiovascular diseases) at higher rates than male Veterans [8]. Finally, female Veterans of the Gulf War era have a higher prevalence of symptom-based conditions including chronic fatigue syndrome, fibromyalgia, irritable bowel syndrome, and migraines, suggesting higher burden of symptom-based conditions [9]. More specifically, female Veterans with GWI had more pronounced autonomic nervous system (ANS) distinctions in comparison to male Veterans on an electrocardiogram test [10], however, additional autonomic research found comparable rates between female and male Veterans with GWI [11]. Yet, a preliminary analysis found that female Gulf War Veterans with GWI had increased mortality rates from digestive system diseases in comparison to non-deployed female Veterans of the same era [12, 13].

Some investigations have found that female Gulf War era Veterans have utilized more health services over men. One study found that female Veterans of the Gulf Era reported more doctor/clinic visits [9]. Another study found that women who served in the Gulf War utilized more outpatient services, inpatient services, and Veterans Affairs (VA) compensation in comparison to men [14]. Notably, certain groups (i.e., older individuals, those hospitalized before war) of Gulf War female Veterans had higher risk of hospitalizations despite cause [15, 16].

Researchers compared groups based on gender and illness group (i.e., GWI, Chronic Fatigue Syndrome (CFS), and healthy control) and found support for gender specific profiles based on differential immune expression [17]. When investigating GWI and CFS in females, Smylie et al. [17] found that interleukin 10 (IL-10) delineated GWI or CFS subjects in females in the context of the interleukin 23 (IL-23)/interleukin 17 (IL-17) axes. Furthermore, the IL-23/IL-17 axis was implicated in GWI expression with sex-specific markers, suggesting that sex hormones modulate the immune response. Researchers have also argued for additional research investigating differential immune expression given that many female Veterans of the Gulf War are developing menopausal symptoms. Therefore, menopause could contribute to cytokine expression, particularly in pro-inflammatory cytokines such as interleukin 1 (IL-1), interleukin 6 (IL-6), and tumor necrosis factor alpha (TNF-alpha; [18]).

Previous investigations have established that deployment to the Gulf War is associated with decreased functioning and poorer health overall in conjunction with a higher prevalence of physical and mental conditions [19–21]. However, research on the impact of the Gulf War for female Veterans remains limited, particularly when investigating mental health outcomes. One of the few studies found that female Gulf War Veterans were more likely than their male counterparts to test positive on a screen for psychological disorders (i.e., major depressive disorder, anxiety, post-traumatic stress disorder (PTSD); [9]).

Focusing on PTSD in specific, Wolfe et al. [22] found female Veterans of the Gulf War were at higher risk of developing PTSD than their male counterparts. Smith et al. [23] found that post-trauma symptoms mediated the relationship between sexual harassment and assault during deployment and military sexual trauma and physical symptoms (gastrointestinal, genitourinary, musculoskeletal, and neurological). Previous research has also established that PTSD or stress-related symptoms have an impact on Gulf War Veterans despite the lower prevalence rate [1]. PTSD, a more severe presentation after trauma exposure, is associated with poor quality of life from health consequences [24] as well as higher endorsement of physical symptoms [25, 26]. Even exposure to traumatic events was linked to more mental health services utilized [27]. However, once again, research in trauma specific to female Gulf War Veterans remains sparse.

Research into PTSD symptoms and stressors of female Gulf War Veterans has investigated combat trauma as well as non-military sources of trauma (i.e., interpersonal distress, sexual trauma). A study by Wolfe et al. [28] found that sexual assault increased PTSD symptoms moreso than combat suggesting that experiencing sexual assault is more detrimental than combat exposure. Furthermore, sexual harassment was predictive of PTSD symptoms outcome. Vogt, Pless, King and King [29] found that female Veterans endorsed more interpersonal stressors than their male counterparts, and these had a stronger impact on their mental health. Another study by Rosen and colleagues [30] found that anticipation of combat was a significant stressor for female Veterans of the Gulf War and was identified as a significant predictor of increased psychological symptoms. However, Sutker and colleagues [31] reported that women were not necessarily more vulnerable to psychological distress when compared to Veterans belonging to an ethnic minority group. These findings seem to suggest that the external environmental stress factors which intersect with sex (and which are similarly experienced by minority groups) rather than sex alone are responsible for these results, rather than inherent biological differences. Further investigation in this area is needed to make clear delineations.

As both PTSD and GWI have been linked to poor health and mental outcomes in women who served in the Gulf War, this study aimed to investigate both the unique and combined influence of PTSD and GWI on self-reported measures of health functioning, and fatigue. Following the GWI Common Data Elements (CDE) [32] we specifically focus on the Centers for Disease Control and Prevention (CDC) as a core definition of GWI, the RAND Short Form 36 item Health survey (SF-36) as it is a core element measure of quality of life, and the multidimensional fatigue inventory (MFI) as it is a highly recommended supplemental measure of the fatigue subgroup of GWI. The fatigue subgroup was specifically focused on because abnormal fatigue is one of the most prevalent and debilitating symptoms of GWI [33], and diagnosis of PTSD is strongly associated with severe fatigue [34, 35]. The analyses investigated potential trends in grouping female Veterans on measures of quality of life, fatigue, PTSD, and GWI status. The overall hypothesis is that female Veterans with combined GWI and high PTSD symptoms will endure worse quality of life and greater fatigue compared to female veterans with GWI and low PTSD symptoms, as well as compared to healthy civilian controls. Although this study is novel in its investigation of functional consequences, it is expected that female Veterans will have similar trends to male Veterans in that a combined presentation of GWI with PTSD symptoms will have worse consequences [36].

## Methods

### Ethics statement

All participants signed an informed consent approved by the Institutional Review Board (IRB) of the Miami VA Medical Health Center (MVAMHC), the University of Miami and/or the IRB of Nova Southeastern University. Ethics review and approval for data analysis was also obtained by the IRB of Nova Southeastern University. All methods were carried out in accordance with relevant guidelines and regulations.

### Cohort

Subjects were recruited from within the clinic population seen in the MVAMHC and university clinics directed by Dr. Klimas via advertisements, doctor’s offices, and community settings (churches and community centers). Depending on the length and number of assessments (i.e. self-report measures, cognitive assessments, exercise challenge, blood draws) subjects were compensated between $50 and $100 per visit for their time and travel costs at the time that they completed each visit. The research cohort was recruited under two VA merit awards, I01CX000205-01 (Klimas PI; GWI: *n* = 7, Healthy Controls (HC): *n* = 6), and I01CX001050-01A1 (Klimas PI; GWI: *n* = 27), a National Institutes of Health R01 award 5R01NS090200-02 (Fletcher PI; HC: *n* = 29), and a Department of Defense Congressionally Directed Medical Research Program award W81XWH-09-2-0071 (Klimas PI; GWI: *n* = 1). Therefore, the full sample for the univariate analyses included 35 female Veterans with GWI and 35 healthy controls. The demographic factors are presented in Table 1. The sample consisted of 42.9% Black, 1.4% Asian, 1.4% Pacific Islander/Native American, 40% White, and 14.3% White Hispanic. All participants were female with an average age of 51.51 years with an average BMI of 27.98. Inclusion criteria for GWI participants were based on the CDC definition of GWI derived from Fukuda et al. [37] to identify Veterans deployed to the theater of operations between August 8, 1990, and July 31, 1991. Veterans in the GWI group endorsed the presence of one or more symptoms for 6 months from at least 2 of the following categories: fatigue; mood and cognitive complaints; and musculoskeletal complaints. The CDC definition captures the three symptoms commonly reported in the literature but is broad and inclusive (especially the mild–moderate form) [38], which results in a high prevalence rate where ~ 50% of Gulf War Veterans would be classified as GWI cases when no exclusionary criteria are defined [39]. The CDC definition of GWI has been found to be useful in clinical settings to rule out disease [40], was supported by Collins et al. [41] for use in controlled clinical trials, and has been recommended by the Institute of Medicine (IOM) for use in clinical practice [38]. This definition has been the most commonly used and is accepted internationally [42, 43]. For this study participants had to meet the CDC GWI case definition in addition to endorsing being in good health prior to 1990 and having no current exclusionary diagnoses defined by Reeves et al. [44] including exclusion of major dementias of any type, alcoholism or drug abuse, medical conditions including organ failure, rheumatologic disorders, and use of medications that impact immune function, such as steroids or immunosuppressives. While female Gulf War era healthy veteran controls are the gold standard comparator, the small percentage of subjects in the recruitment area meeting healthy criteria without exclusionary diagnoses made recruitment of this group unfeasible. As such, control participants consisted of age and BMI matched female civilians self-defined as healthy with no exclusionary diagnoses, and sedentary (no regular exercise program, sedentary employment) to match the activity levels of the subjects with GWI.

**Table 1:** Cohort demographics

Group	Total	GWI	GWI+ *	GWI− *	HC	p_2_	p_3_
N	70	35	19	16	35		
Mean age (y)	48.23 ± 1.08	51.29 ± 1.28	49.79 ± 1.46	53.06 ± 2.17	51.74 ± 1.47	0.818	0.486
Mean BMI	27.43 ± 0.56	28.75 ± 0.87	27.99 ± 1.16	29.61 ± 1.32	26.99 ± 0.91	0.167	0.263
Race/Ethnicity						0.213	0.182
Asian	1.4%	0.0%	0.0%	0.0%	2.9%		
Black	42.9%	54.3%	63.2%	43.8%	31.4%		
White Hispanic	14.3%	11.4%	15.8%	6.3%	17.1%		
White	40.0%	31.4%	15.8%	50.0%	48.6%		
Other	1.4%	2.9%	5.3%	0.0%	0.0%		
Marital status						0.325	0.532
Married	28.6%	28.6%	21.1%	37.5%	34.3%		
Widowed	4.3%	8.6%	10.5%	6.3%	0.0%		
Divorced	31.4%	34.3%	31.6%	37.5%	28.6%		
Never married	10.0%	5.7%	10.5%	0.0%	14.3%		
Not Answered	24.3%	22.9%	26.3%	18.8%	22.9%		
Employed	41.4%	31.4%	42.1%	18.8%	51.4%	0.089	0.089
Education						0.516	0.527
High School	32.9%	28.6%	21.1%	37.5%	37.1%		
College	47.1%	48.6%	52.6%	43.8%	45.7%		
Not answered	20.0%	22.9%	26.3%	18.8%	17.1%		

*GWI+: GWI with PTSD symptoms; GWI−: GWI without PTSD symptoms

### Measures

All participants received a physical examination and medical history including the GWI symptom checklist as per the case definition and completed the SF-36 [45, 46], MFI [47], and the Davidson trauma scale (DTS; [48]) questionnaires.

The SF-36 [45, 46], a health-related quality of life assessment with eight resulting composite scores (physical functioning, role limitations due to physical problems, general health perceptions, energy/fatigue (vitality), social functioning, role limitations due to emotional problems, emotional well-being and pain), was used as it is a core measure of the GWI CDE [32]. It is a widely validated instrument with internal consistency (Cronbach’s alpha) ranging from 0.81 to 0.90 for the SF-36 subscales, with no meaningful differences across deployment status, excellent test–retest agreement (90–97%), and weighted kappa statistics from 0.39 to 0.79 for Gulf War era veterans in specific [49].

The MFI [47], a 20-item self-report instrument designed to measure fatigue with five resulting composite scores (physical fatigue, mental fatigue, reduced activity, reduced motivation, and general fatigue), was used as it is a highly recommended supplemental measure of fatigue in GWI [32]. The measure is not yet rated in terms of validity and reliability specifically for GWI, however it has been shown to have average inter-item correlations from 0.38 to 0.61 indicating no item redundancy, corrected item-total correlations greater than 0.30, Cronbach's alpha coefficients at a level of 0.70, and no significant floor/ceiling effects for chronically unwell fatiguing illnesses such as the GWI related illness CFS [50].

The DTS [48], a self-reporting and rating questionnaire that measures the frequency and severity of DSM-IV symptoms of PTSD within three specific clusters (intrusion, avoidance/numbing, and hyperarousal), was used to assess PTSD symptoms as it is a highly recommended supplemental neuropsychological measure in the GWI CDE [32]. Clusters are scored separately, and PTSD probability is determined through the total scores as well as individual ratings. The total scores are reflective of the frequency and severity ratings of all 17 items within the DTS. According to McDonald et al. [51] a simple cut off score of 70 as the total DTS score has shown effectiveness in predicting PTSD diagnosis in veterans with a 90% classification accuracy rate in all cases based on Kraemer's *kappa,* and provided an accurate estimate of PTSD population prevalence (12–13%).

### Data analysis

#### Hierarchical multiple regression analysis

Investigators utilized univariate hierarchical regression analysis to investigate if GWI co-morbid with higher PTSD symptom load predicted worse outcomes as determined by self-report measures of quality of life and fatigue. The hierarchical regression analyses were conducted in MATLAB v2021a using the *stepwiselm* function to investigate the impact of GWI and PTSD symptoms on self-reported levels of health parameters (RAND SF-36) and fatigue (MFI). The first block (Model 1) contained the categorical variable of health condition as defined by either a healthy control or a participant with GWI. The second block (Model 2) was comprised of the continuous variable of the total DTS score giving a measure of overall PTSD symptoms. These two blocks were assessed to find the contribution to the Coefficient of Determinization (*R*^2^) for the eight scales of the RAND-36 and the five subscales of the MFI. All analyses were interpreted with the alpha level of 0.05. Effect sizes for GWI and PTSD symptom levels were interpreted using multiple *R*^2^ change cutoffs as determined by Cohen [52] with a negligible effect being smaller than 0.02, a small effect being above 0.02, a medium effect being above 0.13, and a large effect being 0.26 or higher. Finally, missing data was minimal (maximum percentile missing = 3.9%) and coded as missing.

#### Cut-point analysis

The application of a cut point at a DTS of 70, as according to McDonald et al. [51], enabled us to stratify GWI subjects establishing individuals with DTS scores 70 and above as probable PTSD positive (GWI+; n = 19), while individuals below 70 were considered as probable PTSD negative (GWI−; n = 16). Quality of life and fatigue measures between the assigned groups were then compared using two sample t-tests with unequal variances. The linear step-up procedure introduced by Benjamini and Hochberg was applied to correct multiple comparisons and ultimately control the false discovery rate [53]. Effect size for the difference between each group was also considered and interpreted in the following ranges [54]: negligible, lower than 0.01, very small, 0.01–0.20, small 0.20–0.50, medium 0.50–0.80, large 0.80–1.20, very large 1.20–2.00, and huge 2.00 or higher. Values were estimated through the corrected Hedges g* equation,

$${g}^{*}= \left(1-\frac{3}{4\left({n}_{1}+{n}_{2}\right)-9}\right)\frac{{\overline{x} }_{1}-{\overline{x} }_{2}}{{s}^{*}}$$

where x̄ was defined as the mean value of the variable for a group, *n* the size of the group, and s* the pooled standard deviation for the variable further defined as,

$${s}^{*}=\sqrt{\frac{\left({n}_{1}-1\right){s}_{1}^{2}+\left({n}_{2}-1\right){s}_{2}^{2}}{{n}_{1}+{n}_{2}-2}}$$

where *s*^2^ is the group variance for the variable. The purpose of utilizing the corrected Hedges g^*^ was to ensure any bias within the population effect size was accounted for, and to overall provide better estimates for smaller sample sizes.

## Results

### Demographics

Demographics for the cohort are given in Table 1. Statistical comparisons were made between both GWI and HC (p_2_), as well as between the GWI+, GWI−, and HC (p_3_) using ANOVA for continuous variables and the χ^2^ test for categorical variables. No statistical differences were found in age, BMI, racial representation, marital status, or education level. While the employment status of the groups did not reach a statistical difference (*p* < 0.05) there was a trend (*p* < 0.089) that GWI participants were less employed compared to HC, with GWI− participants showing the lowest overall employment rate.

### Hierarchical multiple regression

In the hierarchical regression analysis, the quality of life and fatigue measures were each analyzed separately. Predictor blocks were held constant across each measure: Model 1 (GWI or Healthy Control) and Model 2 (PTSD Symptoms). Hierarchical regression were performed with the two blocks of predictor variables to assess how these variables changed the outcome on each of the eight scales of the RAND SF-36, and the five scales of the MFI. A summary of the increase in the coefficients of determination (*R*^2^) of the symptom measures for each regression is presented in Table 2.

**Table 2:** Hierarchical multiple regression model changes in the coefficient of determination for GWI health status (Model 1), and with DTS total score (Model 2)

Measure	Model 1	Model 2
	*ΔR*^2^	*ΔR*^2^
RAND SF-36		
Physical functioning	0.672****	0.024
Physical role^a^	0.630****	0.017
General health perceptions	0.670****	0.038
Energy/fatigue (vitality)	0.652****	0.047
Social functioning	0.601****	0.053
Emotional role^b^	0.632****	0.091**
Emotional well-being	0.458****	0.140***
Pain	0.433****	0.080*
MFI		
Physical fatigue	0.536****	0.059
Mental fatigue	0.569****	0.011
Reduced activity	0.408****	0.097**
Reduced motivation	0.416****	0.005
General fatigue	0.415****	0.094**

*p < 0 .1, **p < 0.05, ***p < 0.01, ****p < 0.001

^a^Physical role (role limitations due to physical health)

^b^Emotional role (role limitations due to emotional problems)

Within Model 1, GWI health status had a significant positive contribution to the variation on all measures within the RAND SF-36 as well as MFI scales. More specifically, some of the largest significant contributions were observed for measures of Physical Functioning and General Health Perceptions from the RAND SF-36 scales with a *R*^2^ change of 0.672 and 0.670 respectively. Model 2 addressed the contribution of DTS scores (PTSD symptoms) in addition to the GWI health status for all measures. PTSD symptoms appeared to have the largest significant contribution to Emotional Well-Being, Role Limitations due to Emotional Problems, and Pain within the RAND SF-36 measures with *ΔR*^2^ of 0.140, 0.091, 0.080, respectively, beyond the contribution of GWI health status. Within the MFI scale measures Reduced Activity and General Fatigue were also significantly increased by the presence of past trauma at increases of 0.097 and 0.094, respectively. While all other measures were increased due to PTSD symptoms, they did not satisfy the criterion for significance. Overall, GWI status had the greatest effect for all symptom reporting, with PTSD symptoms exacerbating all measures.

### Cut-point analysis

**Fig. 1:**
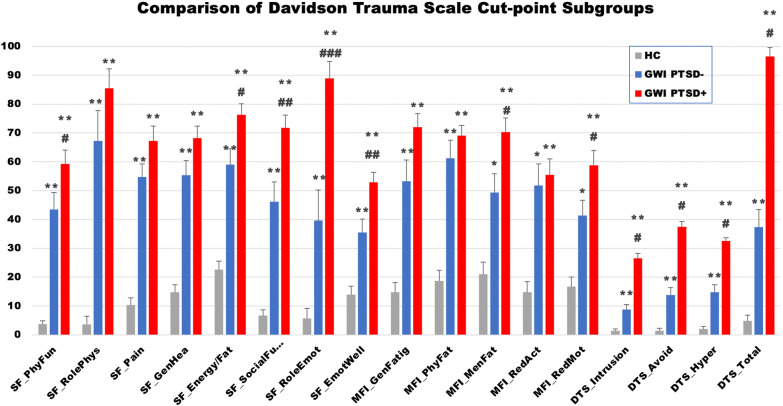
Comparison of symptom scales, and PTSD symptom level scores between Davidson trauma scale cut-point defined groups. *Note*: SF-36 scores shown as (100 − score) to invert scale to align with MFI and DTS such that higher values indicate greater disability. SEM error bars. *p < 0.01; **p < 0.001 as compared to HC via heteroscedastic two sampled t-test, ^#^*p* < 0.05; ^##^*p* < 0.01; ^###^*p* < 0.001 as compared to GWI via heteroscedastic two sampled t-test

As shown in Fig. 1 and Table 3, GWI− and GWI+ had significantly higher values in all measures of the SF-36, MFI, and the DTS scales compared to healthy controls. A further analysis within the GWI subgroups indicated an overall trend in which GWI+ had higher values for all measures compared to GWI−. More specifically, we observed significantly higher values for GWI+ within SF-36 Physical Functioning, SF-36 Energy/Fatigue, SF-36 Social Functioning, SF-36 Role Limitations due to Emotional Problems, SF-36 Emotional Wellbeing, MFI Mental Fatigue, and MFI Reduced Motivation. Furthermore, an understanding of the effect differences showed that GWI− and GWI+ had large or greater effect differences compared to HC for all measures (Table 3). The data also indicated that GWI+ had higher effect differences present in all measures compared to HC than did GWI−. The presence of PTSD symptoms in GWI over GWI alone showed a small effect on MFI Reduced Activity and MFI Physical Fatigue, a medium effect on SF-36 Role Limitations due to Physical Problems, SF-36 Pain, MFI General Fatigue and MFI Reduced Motivation, a large effect on SF-36 Physical Functioning, SF-36 General Health Perceptions, SF-36 Energy/Fatigue, SF-36 Emotional Well-being and MFI Mental Fatigue, and very large effect on SF-36 Social Functioning and SF-36 Role Limitations due to Emotional Problems.

**Table 3:** Comparison of Davidson trauma scale cut-point derived GWI subgroups

Measure	Mean (SEM)			p^c^			g*		
	HC	GWI−	GWI+	HC/GWI−	HC/GWI+	GWI−/GWI+	HC/GWI−	HC/GWI+	GWI−/GWI+
RAND SF-36*									
Physical functioning	3.7 (5.0)	43.4 (5.9)	59.2 (4.8)	< 0.001	< 0.001	0.047	3.06	4.76	0.94
Physical role^a^	3.6 (7.9)	67.2 (10.6)	85.5 (6.7)	< 0.001	< 0.001	0.156	2.27	3.64	0.50
Pain	10.3 (5.3)	54.7 (4.6)	67.2 (5.1)	< 0.001	< 0.001	0.078	2.71	3.10	0.59
General health percept.	14.8 (5.1)	55.3 (5.0)	68.2 (4.2)	< 0.001	< 0.001	0.059	3.04	4.26	0.89
Energy/fatigue	22.7 (5.1)	59.1 (5.5)	76.3 (3.8)	< 0.001	< 0.001	0.016	2.46	4.29	1.18
Social funct.	6.6 (5.8)	46.1 (6.9)	71.7 (4.5)	< 0.001	< 0.001	0.004	2.46	5.34	1.43
Emotional role^b^	5.7 (7.6)	39.6 (10.6)	88.9 (5.9)	0.007	< 0.001	< 0.001	1.35	4.78	1.87
Emotional well-being	13.9 (4.0)	35.5 (4.7)	52.9 (3.4)	< 0.001	< 0.001	0.006	1.20	2.33	1.02
MFI									
General fatigue	14.7 (5.6)	53.2 (7.5)	71.9 (4.8)	< 0.001	< 0.001	0.071	1.61	2.78	0.72
Physical fatigue	18.7 (5.4)	61.2 (6.3)	69.0 (3.6)	< 0.001	< 0.001	0.345	1.82	2.48	0.37
Mental fatigue	21.0 (5.5)	49.4 (6.8)	70.3 (4.9)	0.002	< 0.001	0.028	1.10	2.04	0.87
Reduced activity	14.7 (5.3)	51.7 (7.6)	55.5 (5.6)	0.001	< 0.001	0.728	1.46	1.74	0.13
Reduced motivation	16.7 (4.8)	41.3 (5.3)	58.8 (5.1)	0.001	< 0.001	0.038	1.20	2.01	0.78
DTS									
Intrusion	1.4 (2.0)	8.8 (1.8)	26.6 (1.7)	< 0.001	< 0.001	< 0.001	1.46	4.70	2.37
Avoidance/numbness	1.5 (2.8)	13.8 (2.6)	37.4 (1.8)	< 0.001	< 0.001	< 0.001	1.70	5.86	2.49
Hyperarousal	2.0 (2.5)	14.8 (2.5)	32.5 (1.2)	< 0.001	< 0.001	< 0.001	1.79	5.50	2.21
Total	4.8 (7.1)	37.3 (6.1)	96.5 (3.0)	< 0.001	< 0.001	< 0.001	1.96	7.33	3.02

SF-36 scores shown as (100 − score) to invert scale to align with MFI and DTS such that higher values indicate greater disability

The asterisk denotes the corrected Hedges g value

^a^Physical role (role limitations due to physical health)

^b^Emotional role (role limitations due to emotional problems)

^c^False discovery rate calculated by the method of Benjamini and Hochberg [53] for all significant *p* values is < 0.06

## Discussion

The purpose of this study was to investigate the unique and combined contributions of GWI status and PTSD symptom severity on grouping trends as well as on measures of quality of life and fatigue in female Veterans. This study was specifically designed, given the sparse literature on the functional consequences of conditions linked to military service of GW female Veterans. As such, this study aimed to evaluate whether female Veterans could be grouped by these outcomes, and if these conditions lead to worse quality of life and greater fatigue overall, particularly with co-morbid GWI and high burden of PTSD symptom severity.

Given that there is a relationship between post-trauma symptoms, poorer quality of life, and higher physical symptoms [23–26], and we have previously shown that the presence of past trauma increases GWI symptom presentation in men [36], we hypothesized that co-morbid GWI and high PTSD symptom levels lead to higher endorsement of problematic mental and physical health symptoms compared to GWI as a separate stand-alone condition. The hierarchical model examining GWI in the presence of PTSD symptoms was significant for all domains of reported quality of life and fatigue measures. Of note is the influence of GWI diagnosis on all outcome measures, negatively affecting physical, mental, emotional, and social aspects of patients. GWI health status had a large effect on all the outcomes. The level of PTSD symptoms also influenced reported outcomes above and beyond the influence of GWI in all domains. This was especially the case for emotional well-being in which PTSD had a significant effect of medium scale. Role limitations due to emotional problems, pain, reduced activity and general fatigue were also significantly increased at a smaller size. All other measures added a small increase to symptoms although not significantly, expect for role limitations due to physical health, mental fatigue, and reduced motivation which all had non-significant negligible effects. Overall, these results show that the presence of PTSD symptoms increase the symptom burden associated with GWI on a positively correlated scale.

The most likely explanation for the exacerbation of symptom burden in GWI by PTSD is additional burden on the immune system. The symptoms of GWI are consistent with features of chronic sickness behavior, the underlying basis of which is inflammation [17, 55–59]. Somewhat less appreciated is the link between PTSD and inflammatory activity [60–65]. Understanding how illness affects the immune system in GWI and how it may be altered in the context of PTSD is of key importance to improve understanding of the pathobiology of underlying symptoms of GWI, PTSD and the comorbid combination of these illnesses. Further study in this area is clearly warranted.

While trauma increases symptom burden in a continuous manner, subtyping requires a distinct grouping of subjects. Here we used a cut off score of 70 on the total DTS score according to [51] to define high and low trauma GWI groups. The cut-point analysis shows that compared to healthy sedentary civilian controls, women with GWI and a probable negative PTSD diagnosis still present with a huge symptom burden. A probable PTSD diagnosis with GWI has again an effect increasing scores above GWI alone in all measures. The largest effects are seen in the social and emotional measures, however medium sized effects are also noted in pain, physical functioning and problems, fatigue (both physical and mental), energy levels and overall health perceptions. As such, the cut-point analysis demarcates the two GWI groups as distinct from each other, while also being distinct from controls. The difference is that all symptom measures reported in GWI alone are exacerbated further in the presence of past traumatic stress as evidenced by the effect size differences between measures. Nevertheless, GWVs who do not endorse prior exposure to trauma still report GWI symptomatology, worse quality of life and higher measures of fatigue compared to healthy civilian controls marking GWI as a condition onto itself and making it very unlikely that psychological distress alone is the sole underlying cause of GWI symptoms. This distinction is of importance for the management and treatment of GWI as it suggests a focus on symptom management or treatment strategies for GWI in isolation for those with GWI alone, or in addition to PTSD treatment (rather than solely PTSD treatment) for those with comorbid GWI and PTSD. This is further supported by differences in theoretically predicted treatments for GWI− and GWI+ in male veterans [66], with predictions for females pending.

In comparison to male subjects with GWI the finding in female GWI that PTSD symptoms increase overall symptom burden is analogous [36]. However, the findings of the hierarchical regression differ, in that PTSD symptoms in male GWI significantly increased all symptom measures, whereas significant changes for females with GWI were only noted in emotional measures, pain and fatigue. Specifically, in the hierarchical regression analysis presented here for females the majority of variance in each of the quality of life and fatigue measures is captured by GWI health status (Model 1) with less contribution from the addition of PTSD (Model 2). For their male counterparts GWI health status still captured the greatest amount of variance in each measure, but not to the degree seen for here for females, leaving a larger proportion of variance to be explained significantly by the addition of PTSD [36]. One factor that may explain this difference is female GW era veterans in general reporting higher rates of headaches, aches/pains, fatigue, gastrointestinal difficulties, forgetfulness, and concentration problems as a whole compared to male GW veterans, suggesting a stronger correlation between GWI health status and worse quality of life and fatigue measures [2]. Another factor may be a greater contribution from GWI health status in women due to the use of civilian controls as compared to veteran controls as done with males [36].

In the cut point subgrouping both male and female GWI with probable PTSD showed significantly higher values measures of social functioning, role limitations due to emotional problems, emotional wellbeing, and mental fatigue. However, while there appears to be differences in gender with male GWI+ additionally showing significant increases in general fatigue, and female GWI+ showing significant increases in physical functioning, energy/fatigue levels, and reduced motivation compared to their GWI− counterparts, closer inspection reveals that these measures are trending towards significance (*p* < 0.10) in the other gender. This suggests that the GWI+/GWI− subgroup profiles based on the DTS cut-score of 70 are conserved across gender. Further studies such as this with larger sample size can confirm this.

Overall, our current findings support the understanding that comorbid symptoms of GWI and PTSD subsequently result in poorer quality of life and greater fatigue in female Veterans. This study is not without limitations, namely the small sample size utilized within the study and secondary nature of this analysis. Furthermore, the control comparator group used is a cohort of healthy sedentary civilians, as opposed to Gulf War era healthy sedentary veteran controls. While some literature has asserted that Veterans and civilians are fundamentally different groups with different stressors on health and as such quality of life outcomes cannot be properly compared between them, results are mixed [67], as such our results must be considered in this context. Despite these limitations, the data showed significance measures allowing us to conclude with confidence that both GWI and PTSD have a definitive effect on female Veterans. In the future, additional research would benefit from having groups with differential trauma exposure given the multifaceted nature of PTSD present in female Veterans. It would also benefit from using a wider array of measures in the trauma more specific to females. Additionally, as GWI becomes more identifiable through biological profiles, future research investigating biological data (i.e. blood panels, omics assays etc.) particularly those sensitive to toxin exposure and immunological functioning would greatly aid in clarifying the clinical picture of GWI with and without PTSD symptom presentation. Therefore, it is our hope that this research prompts further investigation and helps inform clinicians about additional patterns they may encounter in female Veteran patients with GWI.

## Data Availability

The datasets used and/or analyzed during the current study available from the corresponding author on reasonable request.

## References

[1] Binns J, Bloom F, Bunker J, Crawford F, Golomb B, Graves J (2014). Research advisory committee on Gulf War Veterans' illnesses. Gulf War Illness and the health of Gulf War Veterans: research update and recommendations, 2009–2013.

[2] Sullivan K, Krengel M, Heboyan V, Schildroth S, Wilson CC, Iobst S (2020). Prevalence and patterns of symptoms among female veterans of the 1991 Gulf War era: 25 years later. J Womens Health (Larchmt).

[3] Health NIo (1994). The Persian Gulf experience and health. NIH technology assessment workshop panel. JAMA.

[4] Craddock TJA, Fritsch P, Rice MA, Del Rosario RM, Miller DB, Fletcher MA (2014). A role for homeostatic drive in the perpetuation of complex chronic illness: Gulf War Illness and chronic fatigue syndrome. PLoS ONE.

[5] Bond EF (2004). Women's physical and mental health sequellae of wartime service. Nurs Clin North Am.

[6] Coughlin SS, Krengel M, Sullivan K, Pierce PF, Heboyan V, Wilson LCC (2017). A review of epidemiologic studies of the health of Gulf War women veterans. J Environ Health Sci.

[7] Wallin MT, Culpepper WJ, Coffman P, Pulaski S, Maloni H, Mahan CM (2012). The Gulf War era multiple sclerosis cohort: age and incidence rates by race, sex and service. Brain.

[8] Brown MC, Sims KJ, Gifford EJ, Goldstein KM, Johnson MR, Williams CD (2019). Gender-based differences among 1990–1991 Gulf War era veterans: demographics, lifestyle behaviors, and health conditions. Womens Health Issues.

[9] Dursa EK, Barth SK, Porter BW, Schneiderman AI (2019). Health status of female and male Gulf War and Gulf Era veterans: a population-based study. Womens Health Issues.

[10] Stein PK, Domitrovich PP, Ambrose K, Lyden A, Fine M, Gracely RH (2004). Sex effects on heart rate variability in fibromyalgia and Gulf War Illness. Arthritis Care Res Off J Am Coll Rheumatol.

[11] Haley RW, Charuvastra E, Shell WE, Buhner DM, Marshall WW, Biggs MM (2013). Cholinergic autonomic dysfunction in veterans with Gulf War Illness: confirmation in a population-based sample. JAMA Neurol.

[12] Barth SK, Kang HK, Bullman TA, Wallin MT (2009). Neurological mortality among U.S. veterans of the Persian Gulf War: 13-year follow-up. Am J Ind Med.

[13] Binns J, Barlow C, Bloom F, Clauw D, Golomb B, Graves J (2008). Research advisory committee on Gulf War Veterans' Illnesses. Gulf War Illness and the health of Gulf War Veterans.

[14] Carney CP, Sampson TR, Voelker M, Woolson R, Thorne P, Doebbeling BN (2003). Women in the Gulf War: combat experience, exposures, and subsequent health care use. Mil Med.

[15] Gray GC, Coate BD, Anderson CM, Kang HK, Berg SW, Wignall FS (1996). The postwar hospitalization experience of U.S. veterans of the Persian Gulf War. N Engl J Med.

[16] Smith TC, Gray GC, Weir JC, Heller JM, Ryan MA (2003). Gulf War Veterans and Iraqi nerve agents at Khamisiyah: postwar hospitalization data revisited. Am J Epidemiol.

[17] Smylie AL, Broderick G, Fernandes H, Razdan S, Barnes Z, Collado F (2013). A comparison of sex-specific immune signatures in Gulf War Illness and chronic fatigue syndrome. BMC Immunol.

[18] Coughlin SS (2016). Need for studies of the health of Gulf War women veterans. Mil Med.

[19] Dursa EK, Barth SK, Schneiderman AI, Bossarte RM (2016). Physical and mental health status of Gulf War and Gulf Era veterans: results from a large population-based epidemiological study. J Occup Environ Med.

[20] Kang HK, Li B, Mahan CM, Eisen SA, Engel CC (2009). Health of US veterans of 1991 Gulf War: a follow-up survey in 10 years. J Occup Environ Med.

[21] Iannacchione VG, Dever JA, Bann CM, Considine KA, Creel D, Carson CP (2011). Validation of a research case definition of Gulf War Illness in the 1991 US military population. Neuroepidemiology.

[22] Wolfe J, Erickson DJ, Sharkansky EJ, King DW, King LA (1999). Course and predictors of posttraumatic stress disorder among Gulf War Veterans: a prospective analysis. J Consult Clin Psychol.

[23] Smith BN, Shipherd JC, Schuster JL, Vogt DS, King LA, King DW (2011). Posttraumatic stress symptomatology as a mediator of the association between military sexual trauma and post-deployment physical health in women. J Trauma Dissoc.

[24] Barrett DH, Gray GC, Doebbeling BN, Clauw DJ, Reeves WC (2002). Prevalence of symptoms and symptom-based conditions among Gulf War Veterans: current status of research findings. Epidemiol Rev.

[25] Engel CC, Liu X, McCarthy BD, Miller RF, Ursano R (2000). Relationship of physical symptoms to posttraumatic stress disorder among veterans seeking care for Gulf War-related health concerns. Psychosom Med.

[26] Wachen JS, Shipherd JC, Suvak M, Vogt D, King LA, King DW (2013). Posttraumatic stress symptomatology as a mediator of the relationship between warzone exposure and physical health symptoms in men and women. J Trauma Stress.

[27] Gade DM, Wenger JB (2011). Combat exposure and mental health: the long-term effects among US Vietnam and Gulf war Veterans. Health Econ.

[28] Wolfe J, Sharkansky EJ, Read JP, Dawson R, Martin JA, Ouimette PC (1998). Sexual harassment and assault as predictors of PTSD symptomatology among US female Persian Gulf War military personnel. J Interpers Violence.

[29] Vogt DS, Pless AP, King LA, King DW (2005). Deployment stressors, gender, and mental health outcomes among Gulf War I veterans. J Trauma Stress.

[30] Rosen LN, Wright K, Marlowe D, Bartone P, Gifford RK (1999). Gender differences in subjective distress attributable to anticipation of combat among U.S. Army soldiers deployed to the Persian Gulf during Operation Desert Storm. Mil Med.

[31] Sutker PB, Davis JM, Uddo M, Ditta SR (1995). Assessment of psychological distress in Persian Gulf troops: ethnicity and gender comparisons. J Pers Assess.

[32] Cohen DE, Sullivan KA, McNeil RB, Klimas NG, McNeil R, Ashford W (2021). A common language for Gulf War Illness (GWI) research studies: GWI common data elements. Life Sci.

[33] Mawson AR, Croft AM (2019). Gulf War Illness: unifying hypothesis for a continuing health problem. Int J Environ Res Public Health.

[34] Lerdal A, Lee KA, Rokne B, Knudsen O, Wahl AK, Dahl AA (2010). A population-based study of associations between current posttraumatic stress symptoms and current fatigue. J Trauma Stress.

[35] McCallum SM, Batterham PJ, Calear AL, Sunderland M, Carragher N, Kazan D (2019). Associations of fatigue and sleep disturbance with nine common mental disorders. J Psychosom Res.

[36] Jeffrey M, Collado F, Kibler J, DeLucia C, Messer S, Klimas N (2021). Post-traumatic stress impact on health outcomes in Gulf War Illness. BMC Psychol.

[37] Fukuda K, Nisenbaum R, Stewart G, Thompson WW, Robin L, Washko RM (1998). Chronic multisymptom illness affecting Air Force veterans of the Gulf War. JAMA.

[38] Radhakrishnan K, Hauser ER, Polimanti R, Helmer DA, Provenzale D, McNeil RB (2021). Genomics of Gulf War Illness in US veterans who served during the 1990–1991 Persian Gulf War: methods and rationale for Veterans Affairs Cooperative Study# 2006. Brain Sci.

[39] Steele L (2000). Prevalence and patterns of Gulf War Illness in Kansas veterans: association of symptoms with characteristics of person, place, and time of military service. Am J Epidemiol.

[40] Baker DG, McQuarrie IG, Murray MG, Lund LM, Dashevsky BA, Mendenhall CL (2001). Diagnostic status and treatment recommendations for Persian Gulf War Veterans with multiple nonspecific symptoms. Mil Med.

[41] Collins JF, Donta ST, Engel CC, Baseman JB, Dever LL, Taylor T (2002). The antibiotic treatment trial of Gulf War Veterans' illnesses: issues, design, screening, and baseline characteristics. Control Clin Trials.

[42] Gwini SM, Forbes AB, Sim MR, Kelsall HL (2016). Multisymptom illness in Gulf War Veterans: a systematic review and meta-analysis. J Occup Environ Med.

[43] White RF, Steele L, O'Callaghan JP, Sullivan K, Binns JH, Golomb BA (2016). Recent research on Gulf War Illness and other health problems in veterans of the 1991 Gulf War: effects of toxicant exposures during deployment. Cortex.

[44] Reeves WC, Lloyd A, Vernon SD, Klimas N, Jason LA, Bleijenberg G (2003). Identification of ambiguities in the 1994 chronic fatigue syndrome research case definition and recommendations for resolution. BMC Health Serv Res.

[45] McHorney CA, Ware JE, Raczek AE (1993). The MOS 36-Item Short-Form Health Survey (SF-36): II. Psychometric and clinical tests of validity in measuring physical and mental health constructs. Med Care.

[46] Ware J, Sherbourne C (1992). The MOS 36-item short-form health survey (SF-36): I. Conceptual framework and item selection. Med Care.

[47] Smets E, Garssen B, Bonke BD, De Haes J (1995). The Multidimensional Fatigue Inventory (MFI) psychometric qualities of an instrument to assess fatigue. J Psychosom Res.

[48] Davidson JR, Book S, Colket J, Tupler L, Roth S, David D (1997). Assessment of a new self-rating scale for post-traumatic stress disorder. Psychol Med.

[49] Voelker MD, Saag KG, Schwartz DA, Chrischilles E, Clarke WR, Woolson RF (2002). Health-related quality of life in Gulf War era military personnel. Am J Epidemiol.

[50] Lin JM, Brimmer DJ, Maloney EM, Nyarko E, Belue R, Reeves WC (2009). Further validation of the Multidimensional Fatigue Inventory in a US adult population sample. Popul Health Metr.

[51] McDonald SD, Thompson NL, Stratton KJ, Calhoun PS (2014). Diagnostic accuracy of three scoring methods for the Davidson Trauma Scale among US military Veterans. J Anxiety Disord.

[52] Cohen J (1988). Statistical power analysis for the behavioral sciences.

[53] Benjamini Y, Hochberg Y (1995). Controlling the false discovery rate: a practical and powerful approach to multiple testing. J R Stat Soc Ser B (Methodol).

[54] Sawilowsky SS (2009). New effect size rules of thumb. J Modern Appl Stat Methods.

[55] Broderick G, Ben-Hamo R, Vashishtha S, Efroni S, Nathanson L, Barnes Z (2013). Altered immune pathway activity under exercise challenge in Gulf War Illness: an exploratory analysis. Brain Behav Immun.

[56] Broderick G, Kreitz A, Fuite J, Fletcher MA, Vernon SD, Klimas N (2011). A pilot study of immune network remodeling under challenge in Gulf War Illness. Brain Behav Immun.

[57] Everson MP, Kotler S, Blackburn WD (1999). Stress and Immune Dysfunction in Gulf War Veterans A. Ann N Yo Açad Sci.

[58] Khaiboullina SF, DeMeirleir KL, Rawat S, Berk GS, Gaynor-Berk RS, Mijatovic T (2015). Cytokine expression provides clues to the pathophysiology of Gulf War Illness and myalgic encephalomyelitis. Cytokine.

[59] Alshelh Z, Albrecht DS, Bergan C, Akeju O, Clauw DJ, Conboy L (2020). In-vivo imaging of neuroinflammation in veterans with Gulf War Illness. Brain Behav Immun.

[60] Baker DG, Nievergelt CM, O'Connor DT (2012). Biomarkers of PTSD: neuropeptides and immune signaling. Neuropharmacology.

[61] Neylan TC, Sun B, Rempel H, Ross J, Lenoci M, O'Donovan A (2011). Suppressed monocyte gene expression profile in men versus women with PTSD. Brain Behav Immun.

[62] Plantinga L, Bremner JD, Miller AH, Jones DP, Veledar E, Goldberg J (2013). Association between posttraumatic stress disorder and inflammation: a twin study. Brain Behav Immun.

[63] Miller MW, Lin AP, Wolf EJ, Miller DR (2018). Oxidative stress, inflammation, and neuroprogression in chronic PTSD. Harv Rev Psychiatry.

[64] Kim TD, Lee S, Yoon S (2020). Inflammation in post-traumatic stress disorder (PTSD): a review of potential correlates of PTSD with a neurological perspective. Antioxidants (Basel)..

[65] Michopoulos V, Powers A, Gillespie CF, Ressler KJ, Jovanovic T (2017). Inflammation in fear- and anxiety-based disorders: PTSD, GAD, and beyond. Neuropsychopharmacology.

[66] Arias FJC, Aenlle K, Abreu M, Holschbach MA, Michalovicz LT, Kelly KA (2021). Modeling neuroimmune interactions in human subjects and animal models to predict subtype-specific multidrug treatments for Gulf War Illness. Int J Mol Sci.

[67] Luncheon C, Zack M (2012). Health-related quality of life among US veterans and civilians by race and ethnicity. Prev Chronic Dis.

